# Crystallization of Anhydrous Copper Sulfate From Sulfuric Acid—Ammonium Sulfate Mixtures

**DOI:** 10.6028/jres.068A.029

**Published:** 1964-06-01

**Authors:** Paul M. Gruzensky

## Abstract

The growth of CuSO_4_ crystals from a nonaqueous solvent, composed of (NH_4_)_2_SO_4_ and H_2_SO_4_ is described. Solubility of CuSO_4_ in solvents of varying (NH_4_)_2_SO_4_ to H_2_SO_4_ ratio, at 200 °C, has been determined, as well as the temperature dependence of the solubility in 0.35(NH_4_)_2_SO_4_ — 0.65H_2_SO_4_. Single crystal specimens, weighing up to 150 mg have been obtained.

## 1. Introduction

Anhydrous CuSO_4_, isostructural with orthorhombic ZnSO_4_, is of particular interest because of its antiferromagnetic properties at low temperatures. Fundamental investigations on this compound would be greatly enhanced if single crystal specimens were readily available. Kokkoros and Rentzeperis [1] 1 obtained CuSO_4_ single crystals up to 1 mm in length by evaporation of an aqueous solution obtained by dissolving CuSO_4_·5H_2_O in water and adding H_2_SO_4_. Conditions under which the anhydrous salt may be obtained from an aqueous solution have been described in the early literature [2]. Kreines [3], in an effort to prepare crystal specimens of this salt for magnetic susceptibility and anisotropy studies, dissolved CuSO_4_ in molten (NH_4_)_2_SO_4_; then, by controlling the rate of decomposition of the solvent, he was able to obtain single crystals of CuSO_4_ weighing up to 2 mg. Like many other sulfates, CuSO_4_ undergoes decomposition before the melting point is reached so that growth from the melt under normal laboratory conditions is precluded. The use of a nonaqueous solvent appears to be the most promising approach, and experiments in our laboratory indicate that a sulfuric acid-ammonium sulfate mixture offers some definite advantages over the single components as a solvent for CuSO_4_ single crystal growth.

## 2. Experimental Results and Discussion

Starting reagents used throughout this work were Baker “Analyzed Reagent” grade. The CuSO_4_·5H_2_O was further purified by recrystallization from distilled and demineralized water, then dehydrated by heating in a muffle furnace at 350 °C for 24 hr under a dry nitrogen atmosphere. The anhydrous powdered salt was stored in a dessicator over phosphorous pentoxide.

The sulfuric acid was adjusted to 100 percent composition by adding fuming sulfuric acid to the commercial 96 percent reagent, the freezing point method [4] being used to determine when the 100 percent composition point was reached.

Solvents of various compositions were then prepared by heating a measured quantity of H_2_SO_4_ to 150 °C and adding a weighed amount of (NH_4_)_2_SO_4_ to give the desired composition.

**Figure 1:**
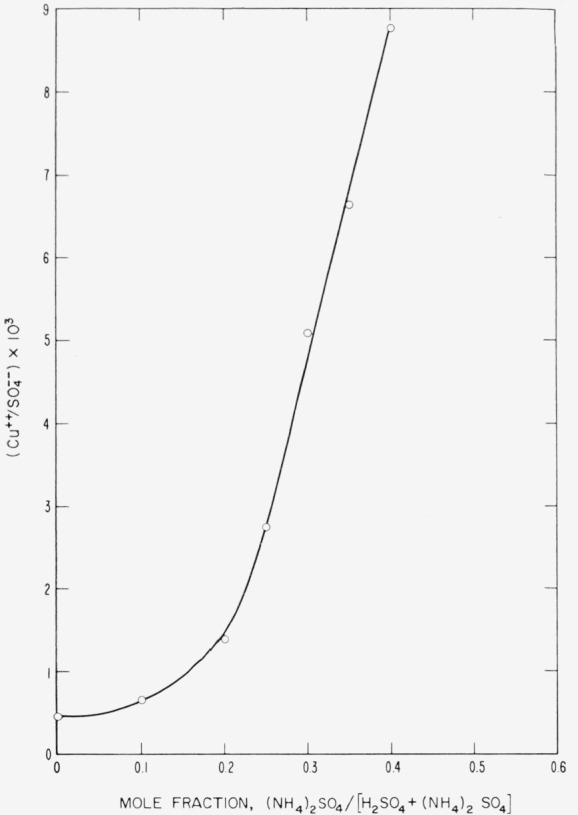
Solubility of *CuSO*_4_ in (*NH*_4_)_2_*SO*_4_–*H*_2_*SO*_4_ of varying composition at 200 °C.

**Figure 2:**
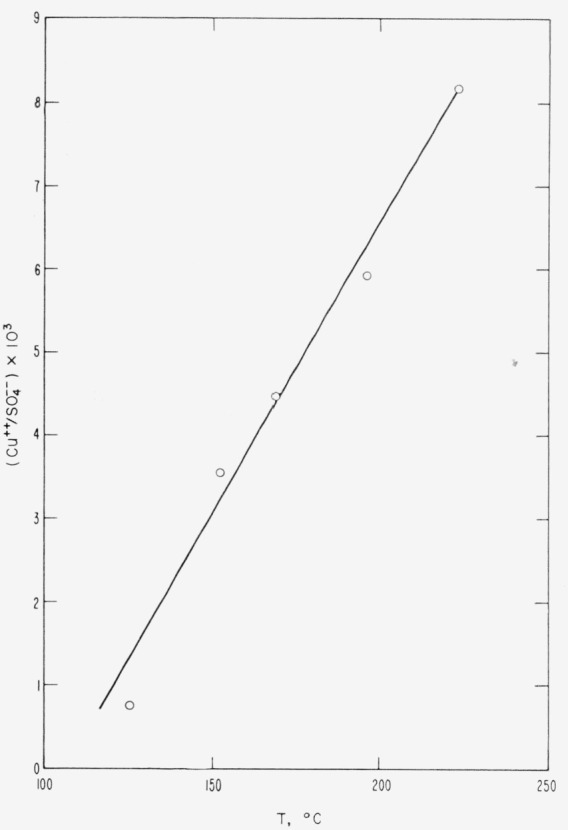
Temperature dependence of *CuSO*_4_ solubility in 0.35(*NH*_4_)_2_*SO*_4_–0.65*H*_2_*SO*_4_.

The solubility of CuSO_4_ in solvents of varying (NH_4_)_2_SO_4_–H_2_SO_4_ ratios, at 200 °C, is indicated in figure 1. The experimental points were determined by adding powdered CuSO_4_ to the solvent, maintaining the temperature at 200 °C ±2 °C for 24 hr to assure equilibrium, sampling the solution, and determining the copper content and sulfate content iodometrically and gravimetrically, respectively. The sulfate analysis was corrected for the amount of sulfate present as CuSO_4_ so that the ordinates of figures 1 and 2 show the ratio of copper to solvent sulfate in the solution.

As figure 1 indicates, the solubility of CuSO_4_ in pure H_2_SO_4_ is relatively low, but increases rapidly as the (NH_4_)_2_SO_4_ content of the solvent increases. However, increasing the (NH_4_)_2_SO_4_ ratio also increases the viscosity of the solvent at any given temperature, so that higher temperatures are necessary to maintain the solvent in a fluid state. Practical working temperatures are limited by the fact that (NH_4_)_2_SO_4_ undergoes considerable decomposition above 300 °C. Since crystal growth is dependent on diffusion through the solution and since higher viscosities affect diffusion adversely, solvents of higher mole ratio than 0.35 (NH_4_)_2_SO_4_ were not considered.

The temperature dependence of CuSO_4_ solubility in 0.35(NH_4_)_2_SO_4_–0.65H_2_SO_4_ is shown in figure 2. In determining the experimental points, the temperature of the solution was controlled at the given value ±2 °C and maintained for 24 hr with occasional stirrings before sampling. A study of the copper content of the solution as a function of time, at the lowest temperature shown in figure 2, indicated that 24 hr was an adequate period to achieve equilibrium. Analyses for copper and sulfate were made as indicated above.

To carry out the crystal growth, approximately 500 ml of solution were placed in a large borosilicate glass test tube, heated by a conventional hot plate. An excess of CuSO_4_ powder was added to the solution, and a tantalum sheet, rotating at 10 rpm, was suspended in the solution near the surface. The test tube was loosely covered. The solution temperature was approximately 200 °C with a temperature gradient between the bottom of the container and the tantalum sheet of approximately 5 °C. Single crystals of CuSO_4_ grew predominantly at the edges of the tantalum sheet, but occasionally also on the flat surfaces. In three or four days, crystals weighing up to 150 mg have been obtained. Excess solvent was removed from the crystal surfaces by washing in 100 percent H_2_SO_4_ and heating to 400 °C.

**Figure 3:**
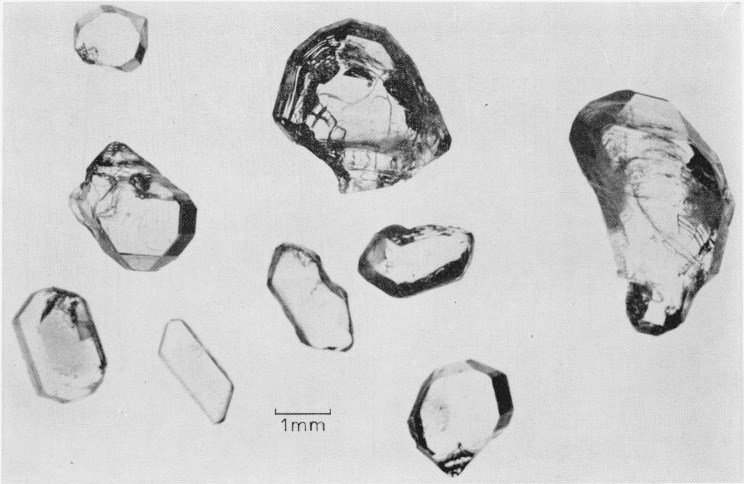
*CuSO*_4_ single crystals.

Figure 3 is a photograph of several CuSO_4_ crystals. The specimens, which were transparent with a slight greenish tinge, were verified to be single crystals by Laue backscatter x-ray diffraction. Chemical analysis of the crystals indicated a composition of 99.7 percent CuSO_4_.

## Footnotes

1 Figures in brackets indicate the literature references at the end of this paper.
